# Supplementary material for: Stem Cell Bioengineering with Bioportides: Inhibition of Planarian Head Regeneration with Peptide Mimetics of Eyes Absent Proteins
Source: Pharmaceutics. 2023 Jul 26;15(8):2018. doi: 10.3390/pharmaceutics15082018 (PMC10458859; doi:10.3390/pharmaceutics15082018)
Supplement: Supplementary file 1 [file pharmaceutics-15-02018-s001.zip › pharmaceutics-2480518-supplementary.pdf]

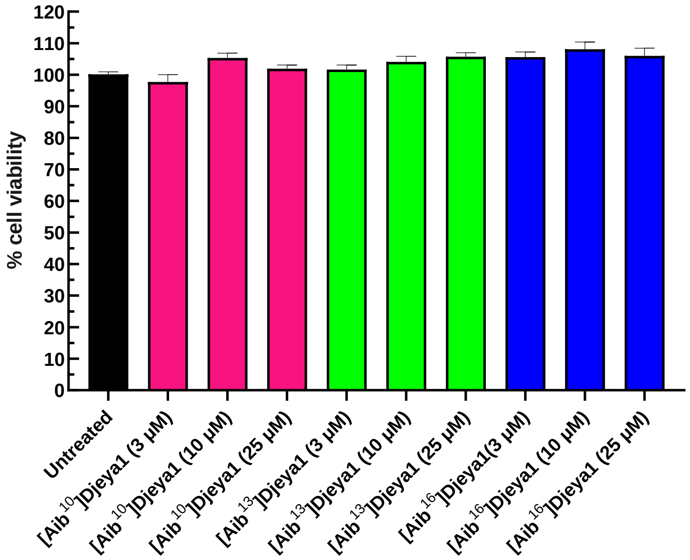

Figure S1. Viability of PC-3 cells treated with Aib-substituted Djeya1 analogues. PC-3 cells were seeded in Roswell Park Memorial Institute media-1640 with L-glutamine (RPMI-1640, Gibco, Life Technologies, USA) growth medium, supplemented with 10% (*v*/*v*) foetal bovine serum (FBS, Gibco, Life Technologies, USA) and 1% (*v*/*v*) penicillin/streptomycin mixture (Gibco, Life Technologies, USA) in 96-well plates (1.00 × 10^4 cells/well) and maintained in an incubator (MCO-170AICUV, Panasonic, Germany) at 37 °C, with a 5% CO_2 atmosphere under fully humidified conditions for 24 h. Then, the cells were incubated with 3, 10 and 25 μM of [Aib^{10}]Djeya1, [Aib^{13}]Djeya1 and [Aib^{16}]Djeya1 in RPMI-1640 medium without FBS and incubated for 24 h. Untreated cells were also included in the assay and used as control (100% viability). One hour before the incubation time, 10 μL of PrestoBlue™ Cell Viability reagent, resazurin-based reagent (Thermo Fisher Scientific, USA) was added to each well and incubated for 1 h at 37 °C and 5% CO_2 atmosphere. After the incubation, 100 μL of the culture medium of each well were transferred to a black bottom 96-well plate and the fluorescence at λ_{abs} = 560 nm and λ_{em} = 590 nm was measured in the microplate reader Tecan Infinite® 200 PROseries, Mannedorf, Switzerland. Cell viability was expressed as the percentage of viable cells, considering untreated cells as 100% viability. Three independent experiments with five replicates for each condition were performed. These data confirm that Aib-substituted Djeya1 analogues do not impact upon the viability of PC-3 cells.

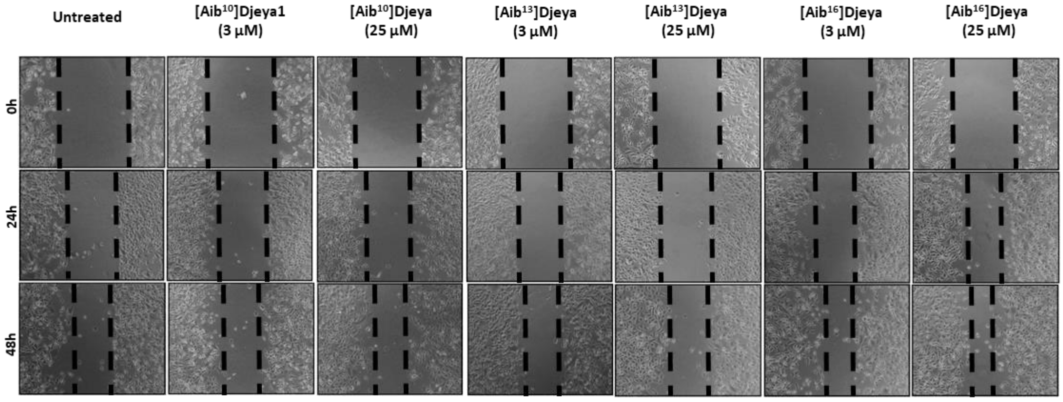

Figure S2. Impact of Aib-substituted Djeya1 peptides (3 μM and 25 μM) on prostate cancer (PC-3) cell migration. This is a representative experiment to evaluate the impact of peptides in the wound healing assay. Wound size was measured at 0, 24, and 48 h.

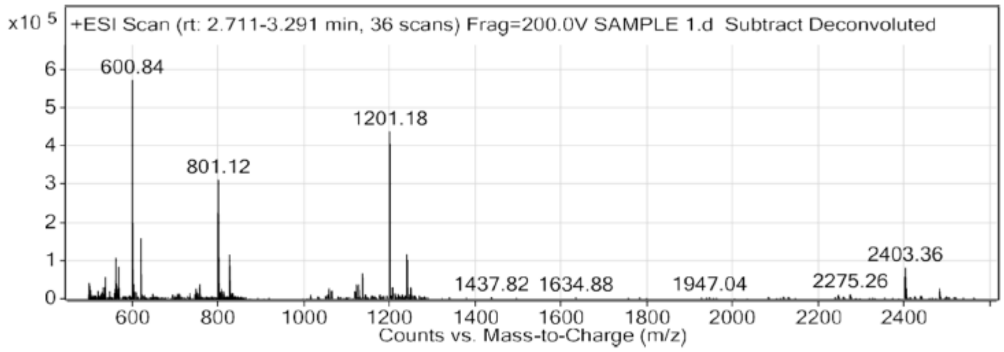

Figure S3. Identification of [Aib^{10}]Djeya 1 by mass spectrometry, Agilent 6200 TOF.

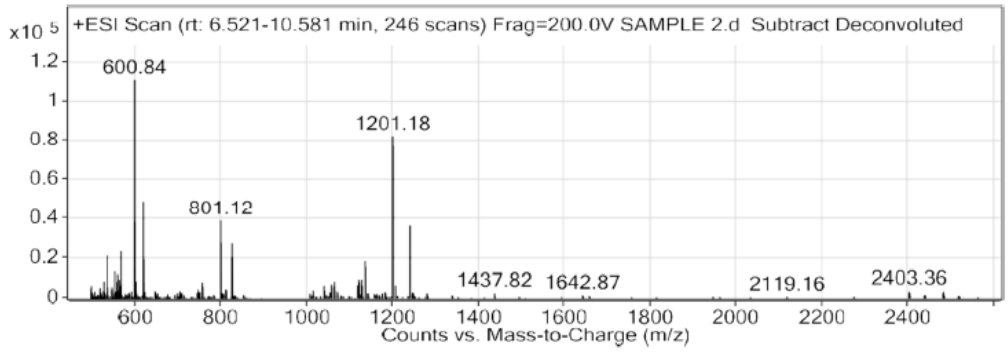

Figure S4. Identification of [Aib^{13}]Djeya 1 by mass spectrometry, Agilent 6200 TOF.

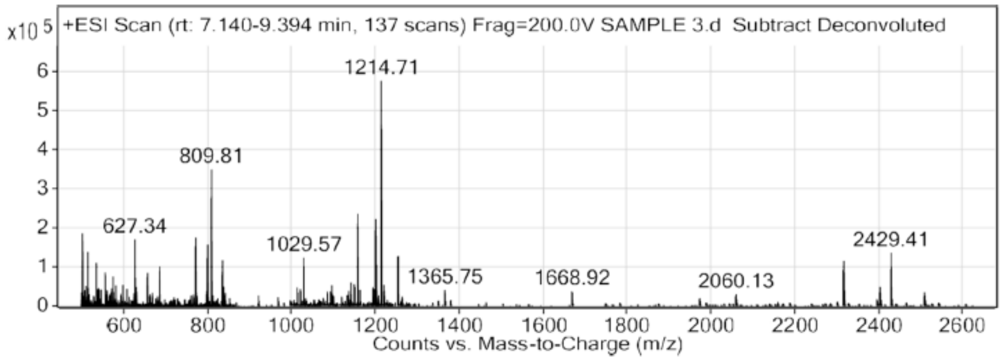

Figure S5. Identification of [Aib^{16}]Djeya1 by mass spectrometry, Agilent 6200 TOF.
